# A212 SYMPTOMS IN PATIENTS WITH NORMAL ESOPHAGEAL MOTILITY MAY DIFFER BASED ON AGE AND SEX

**DOI:** 10.1093/jcag/gwad061.212

**Published:** 2024-02-14

**Authors:** D R Kim, M Woo

**Affiliations:** Medicine, University of Saskatchewan, Saskatoon, SK, Canada; University of Calgary Cumming School of Medicine, Calgary, AB, Canada

## Abstract

**Background:**

High resolution esophageal manometry (HREM) is used to diagnose esophageal motility disorders. While esophageal symptoms including dysphagia are common and seen in up to 20% of adults, a minority of symptomatic patients have esophageal dysmotility, when defined by the Chicago Classification v4 (CCv4). The generation of esophageal symptoms in a patient with normal esophageal motor function is complex, but may relate to visceral hypersensitivity, inappropriate pain perception or unidentified contraction abnormalities. The Esophageal Symptom Questionnaire (ESQ-30) is a validated tool in the assessment of esophageal symptoms and measures the frequency and severity of dysphagia, reflux and globus through three separate sub-scales (ESQ-D, ESQ-R, ESQ-G respectively).

**Aims:**

To determine age and sex in esophageal symptoms (as measured by ESQ-30 sub-scales) in a population of symptomatic patients with normal esophageal motility.

**Methods:**

Retrospective analysis of consecutive reports from HRM studies performed between October 2021 – March 2023 who provided consent for inclusion in the motility databank and completed ESQ-30 questionnaires. All values expressed as median (IQR: 25-75-centile). Spearman’s bivariate correlations were performed between ESQ-D, ESQ-R, ESQ-G and manometric variables (IRP, DCI, % failed, and % weak) as well as demographic variables (age). Group means were compared with the non-parametric Mann-Whitney U test.

**Results:**

Within the study period, 681 patients underwent HREM and had complete ESQ-30 questionnaires. Of these patients, 151 female and 73 male participants (mean age 51.8 +/- 15.2 years) had normal esophageal motility by CCv4. ESQ-D and ESQ-R scores were significantly higher in women in comparison to men. ESQ-D was 10 in women and 5 in men (p=.021), and ESQ-R was 28 in women and 19 in men (p=.029). There was a weak negative correlation between age and reflux symptoms (R^2^ -.139, p = 0.038). There was no significant difference in manometric parameters between males and females (p ampersand:003E .05 for all comparisons). Dysphagia and globus weakly negatively correlated with median IRP (R^2^ -.156, p = .019 and R^2^ -.139, p = .038 for dysphagia and globus respectively).

**Conclusions:**

In a population of symptomatic patients with normal esophageal motility, symptom profile differed based on age and sex. Women had higher dysphagia and reflux symptoms compared to men, while older patients had lower reflux symptoms. These findings suggest that esophageal sensitivity may vary with age and sex in patients with normal esophageal motility.

The finding of higher dysphagia and globus symptoms in patients with lower median IRP (integrated relaxation pressure) is an unexpected finding and may reflect reflux effect. Further studies to assess for the impact of GERD (using 24h pH-metry/MII) on patients with normal esophageal motor function are warranted.

**Funding Agencies:**

None

